# Supplementary material for: Patterns of smartphone typing performance by time awake: implications for unobtrusive ambulatory mental fatigue assessment
Source: PLOS Digit Health. 2026 Mar 26;5(3):e0001281. doi: 10.1371/journal.pdig.0001281 (PMC13020785; doi:10.1371/journal.pdig.0001281)
Supplement: S1 Fig — (DOCX) [file pdig.0001281.s001.docx]

**S1 Fig.** SensorKit Keyboard Usage data collection.

|  | **DATA COLLECTED** | **DATA NOT COLLECTED** |
| --- | --- | --- |
| **SensorKit Keyboard Metrics** | - Length of words - Typing speed - Typing accuracy and the kind of typing errors - Keyboard orientation and screen size - Number of words and emoji typed | - Any of the words typed - Any information about keyboard extensions or stickers |
