# Supplementary material for: Patterns of smartphone typing performance by time awake: implications for unobtrusive ambulatory mental fatigue assessment
Source: PLOS Digit Health. 2026 Mar 26;5(3):e0001281. doi: 10.1371/journal.pdig.0001281 (PMC13020785; doi:10.1371/journal.pdig.0001281)

**S2 Fig.** The numbers of: typing sessions by hours awake (A), subjects by hours awake (B), typing sessions by typing speed (C), and typing sessions by rate of deletion (D) in the sample.


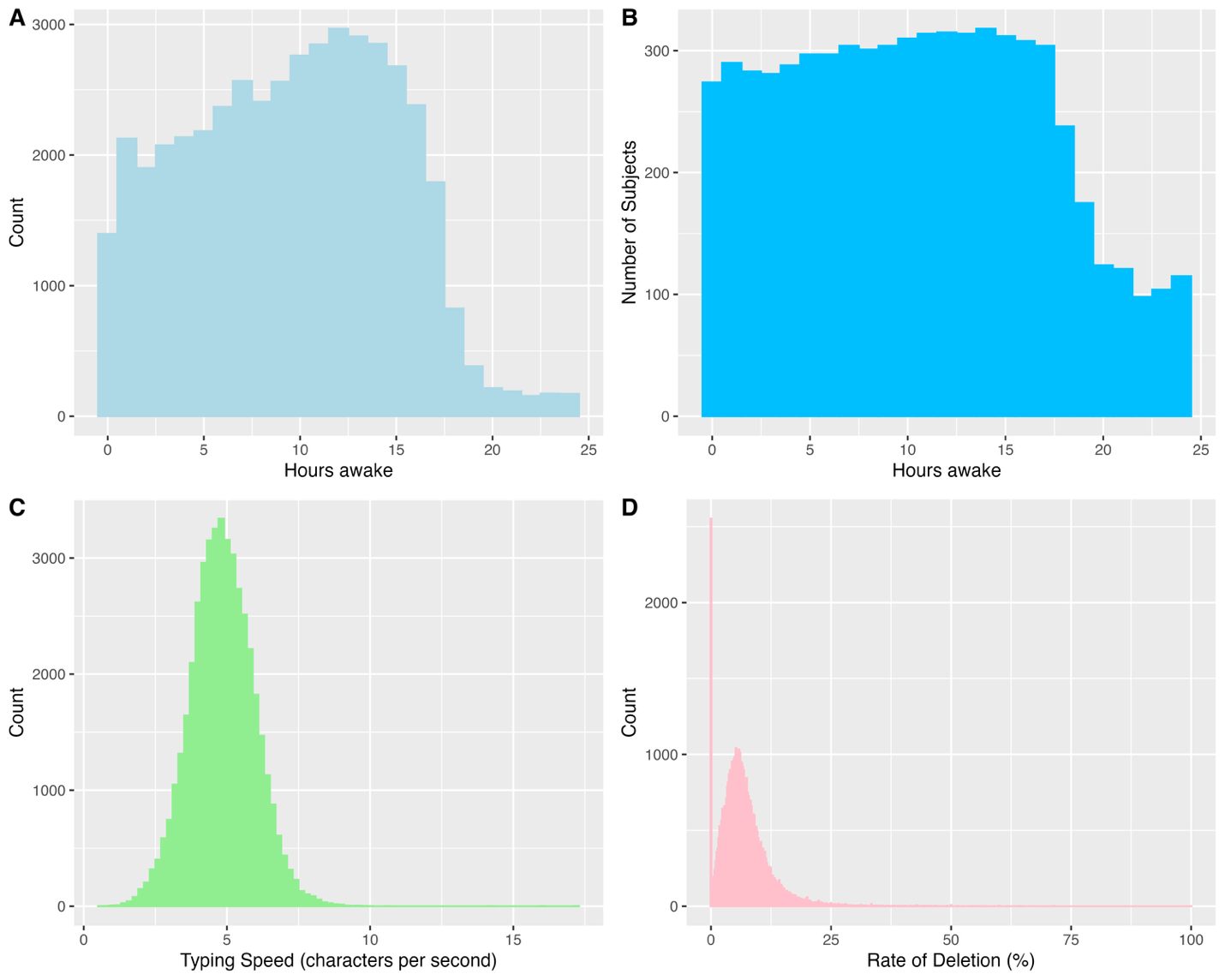

Supplement: S2 Fig — (DOCX) [file pdig.0001281.s002.docx]
