# Supplementary material for: Patterns of smartphone typing performance by time awake: implications for unobtrusive ambulatory mental fatigue assessment
Source: PLOS Digit Health. 2026 Mar 26;5(3):e0001281. doi: 10.1371/journal.pdig.0001281 (PMC13020785; doi:10.1371/journal.pdig.0001281)

**S3 Fig. Average rate of deletion of two example subjects by hours awake.** Rates of deletion were converted to individual z-scores. The Blue solid line represents the individual average (z-score = 0), while the red dashed line represents +0.1 standard deviation above the individual average.


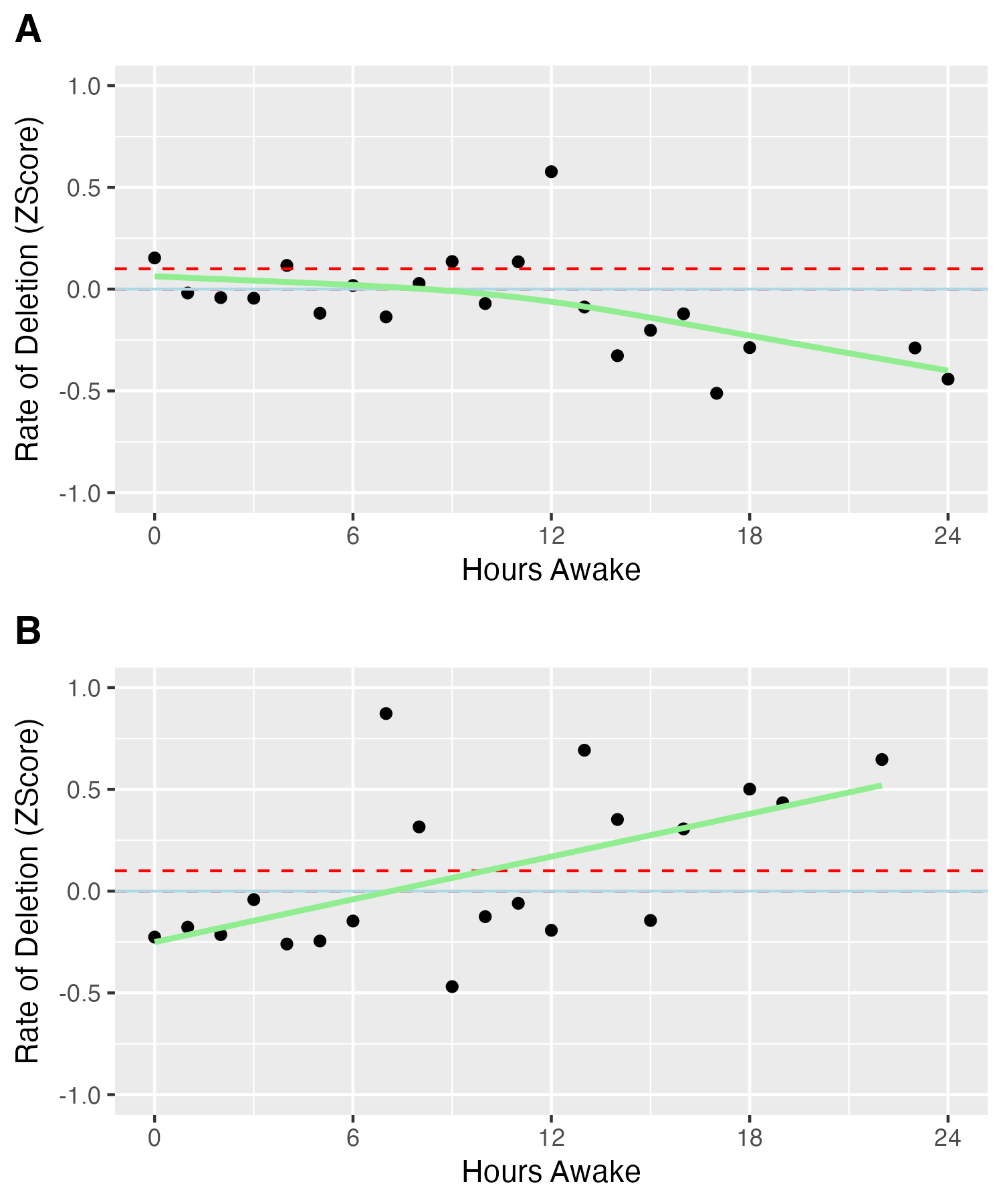

Supplement: S3 Fig — (DOCX) [file pdig.0001281.s003.docx]
