# Supplementary material for: Patterns of smartphone typing performance by time awake: implications for unobtrusive ambulatory mental fatigue assessment
Source: PLOS Digit Health. 2026 Mar 26;5(3):e0001281. doi: 10.1371/journal.pdig.0001281 (PMC13020785; doi:10.1371/journal.pdig.0001281)
Supplement: S5 Fig — (DOCX) [file pdig.0001281.s005.docx]

**S5 Fig. Average typing speed (A) and average rate of deletions (B) by number of hours awake, US English input mode only.** Typing speed and rate of deletions were converted to individual z-scores. The error bars represent 95%CI. The GAM-smoothed prediction lines from model (4) were plotted (green). The Blue solid line represents the individual average (z-score = 0), while the red dashed line represents -0.1 standard deviation in (A) and +0.1 standard deviation in (B).

**
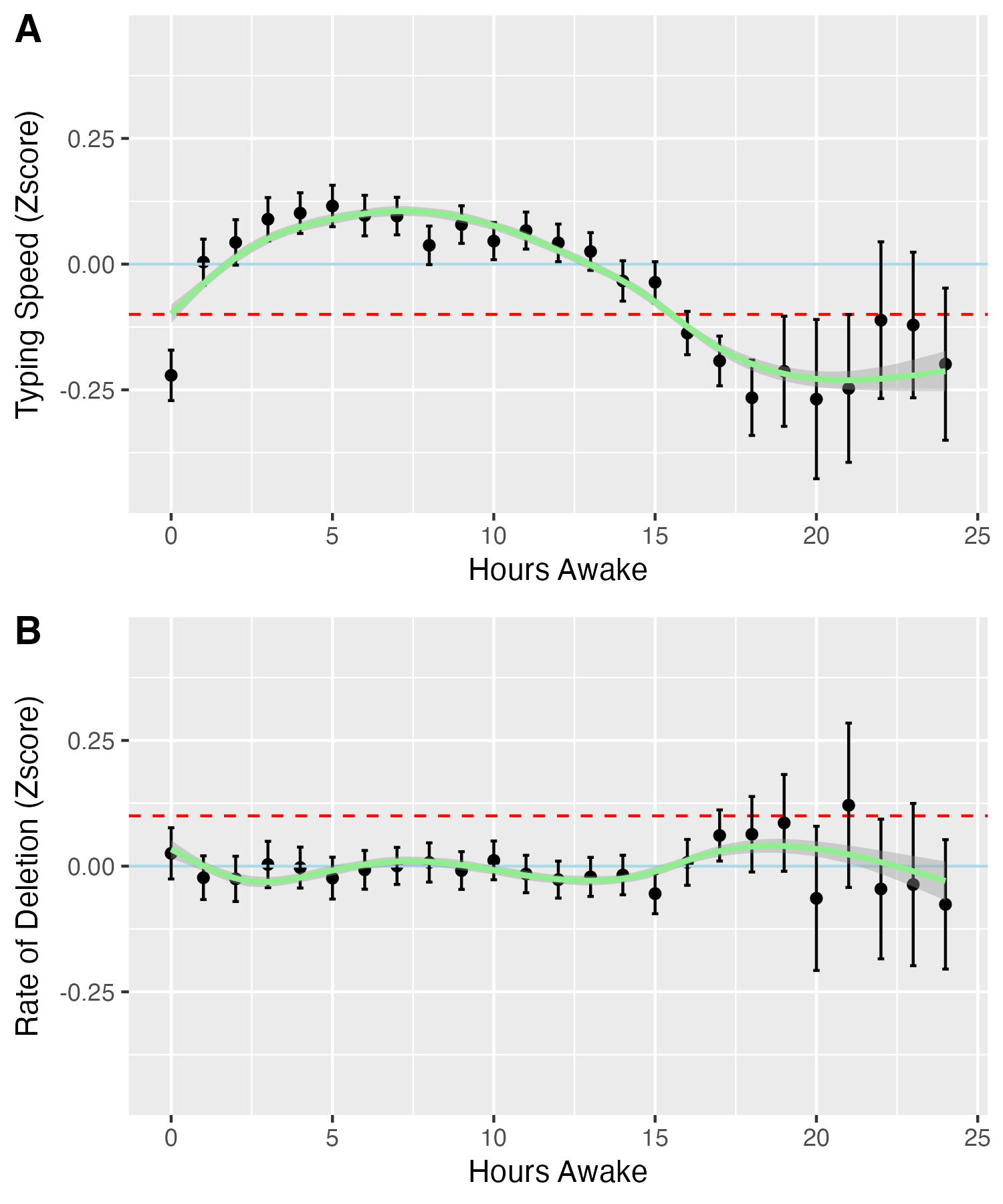
**
