# Supplementary material for: Patterns of smartphone typing performance by time awake: implications for unobtrusive ambulatory mental fatigue assessment
Source: PLOS Digit Health. 2026 Mar 26;5(3):e0001281. doi: 10.1371/journal.pdig.0001281 (PMC13020785; doi:10.1371/journal.pdig.0001281)

**S7 Fig. Average rate of deletion versus hours awake, by the type of wearable device recording sleep.** Rates of deletions were converted to individual z-scores. The error bars represent 95%CI. The GAM-smoothed prediction lines from model (4) were plotted (green). The Blue solid line represents the individual average (z-score = 0), while the red dashed line represents 0.1 standard deviation.


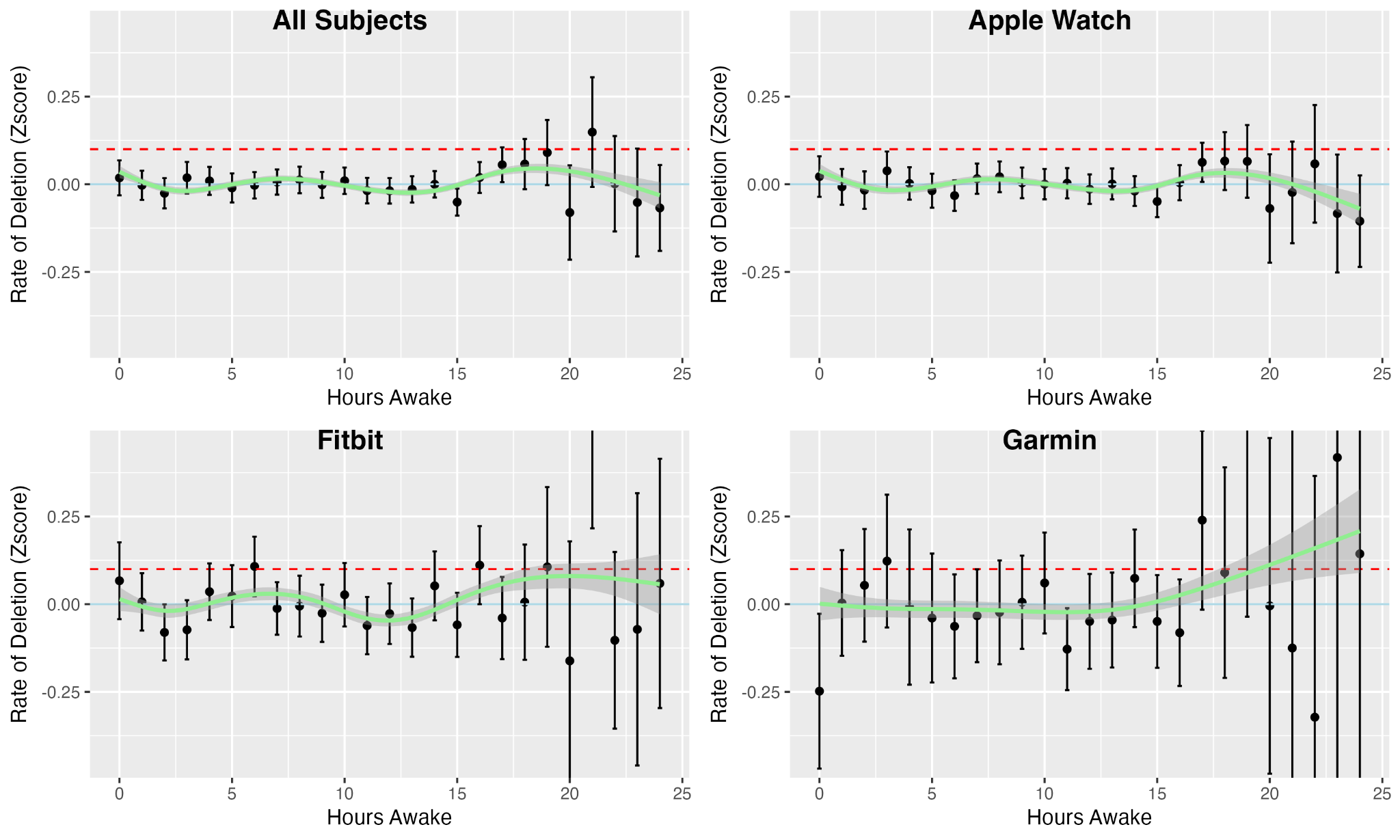

Supplement: S7 Fig — (DOCX) [file pdig.0001281.s007.docx]
