# Supplementary material for: Patterns of smartphone typing performance by time awake: implications for unobtrusive ambulatory mental fatigue assessment
Source: PLOS Digit Health. 2026 Mar 26;5(3):e0001281. doi: 10.1371/journal.pdig.0001281 (PMC13020785; doi:10.1371/journal.pdig.0001281)

**S8 Fig. Proportion of typing sessions with low speed by hours awake in the sample.** Low speed is defined as individual typing speed z-scores<0.1.


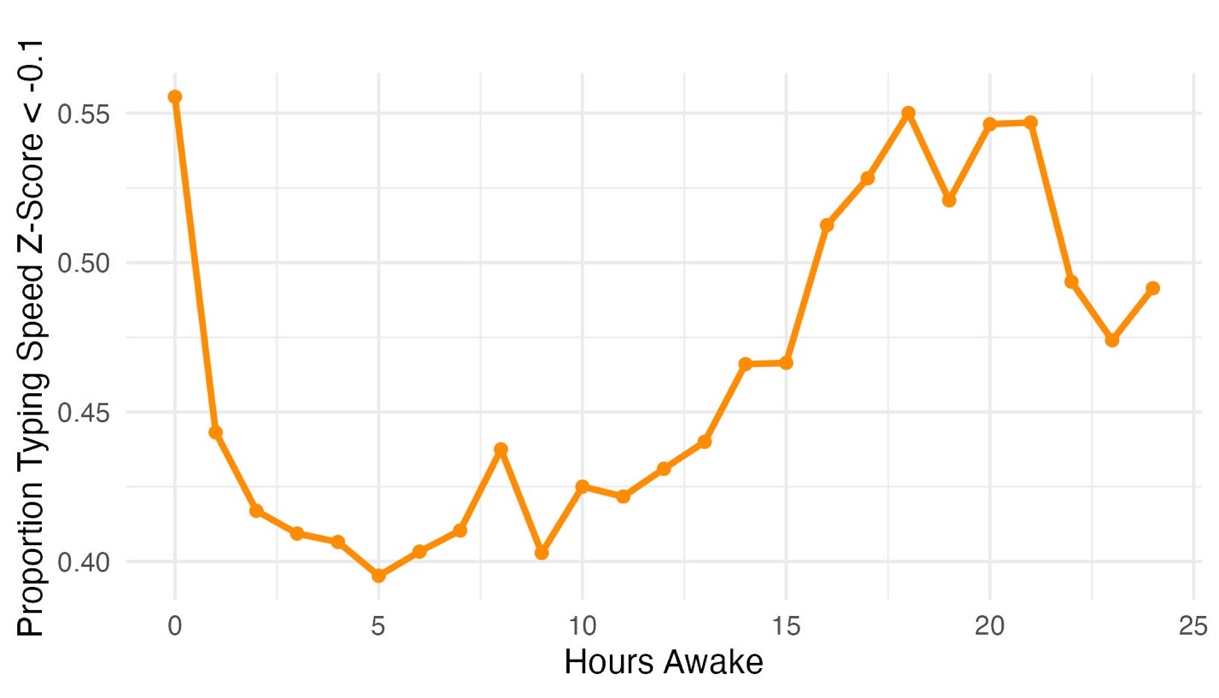

Supplement: S8 Fig — (DOCX) [file pdig.0001281.s008.docx]
