# Supplementary material for: Patterns of smartphone typing performance by time awake: implications for unobtrusive ambulatory mental fatigue assessment
Source: PLOS Digit Health. 2026 Mar 26;5(3):e0001281. doi: 10.1371/journal.pdig.0001281 (PMC13020785; doi:10.1371/journal.pdig.0001281)
Supplement: Attachment — Submitted filename: Response Letter.docx [file pdig.0001281.s010.docx]

4251 Plymouth Rd, Arbor Lakes Bldg. 1
Ann Arbor, MI 48109-3640

January 14, 2026

Dr. Leo Anthony Celi
Editor-in-Chief, PLOS Digital Health

Dr. Tianzhen Chen
Academic Editor, PLOS Digital Health

Dear Drs. Celi and Chen,

Thank you for the opportunity to revise our manuscript, ‘*Patterns of smartphone typing performance by time awake: implications for unobtrusive ambulatory mental fatigue assessment*’, for consideration of publication at PLOS Digital Health. We have revised our submission according to the journal requirements and the reviewers’ comments, which have substantially improved the content and presentation of our manuscript. We have prepared the updated versions of the manuscript and supplemental materials following the resubmission requirements. Below, we respond to the journal requirements and the reviewers’ comments point-by-point.

Thank you for your consideration.

Sincerely,

Zhenke Wu, PhD

Associate Professor of Biostatistics, University of Michigan
Michigan Institute for Data and AI in Society (MIDAS)
Email: [zhenkewu@umich.edu](mailto:zhenkewu@umich.edu)

[zhenkewu.com](http://zhenkewu.com/)

Yu Fang, MSE

Michigan Neuroscience Institute

University of Michigan

Email: [yfang@umich.edu](mailto:yfang@umich.edu)

**Journal Requirements:**

1. We have amended your Competing Interest statement to comply with journal style. We kindly ask that you double check the statement and let us know if anything is incorrect.

The current Competing Interest statement, reading “*The authors have no competing interests that could be perceived to bias this work.*” is correct.

2. Please provide your detailed Financial Disclosure statement. This is published with the article. It must therefore be completed in full sentences and contain the exact wording you wish to be published.

1) Please clarify all sources of funding (financial or material support) for your study. List the grants (with grant number) or organizations (with url) that supported your study, including funding received from your institution.
2) State the initials, alongside each funding source, of each author to receive each grant.
3) State what role the funders took in the study. If the funders had no role in your study, please state: “The funders had no role in study design, data collection and analysis, decision to publish, or preparation of the manuscript.”
4) If any authors received a salary from any of your funders, please state which authors and which funders.

We now added the Financial Disclosure statement (p. 22) before References, and it reads:

“*This research was supported by NIH* [*R01MH101459*](https://www-sciencedirect-com.proxy.lib.umich.edu/science/article/pii/S1389945725005155#gs1) *(to SS, ZW). The funders had no role in study design, data collection and analysis, decision to publish, or preparation of the manuscript. No authors received any salary from any of the funders.*”

3. Please provide separate figure files in .tif or .eps format. For more information about figure files please see our guidelines: [https://journals.plos.org/digitalhealth/s/figures](https://urldefense.com/v3/__https://journals.plos.org/digitalhealth/s/figures__;!!NnSTv5QBqPjS9UMk!LXzi5t0QgzWOMFCm-xWhA2cPfRcZFY-pyeVURkNHnxvvT07RjOxm-G4QSOG-MogUX4ks4KZy_u5hITFMz24$)
[https://journals.plos.org/digitalhealth/s/figures#loc-file-requirements](https://urldefense.com/v3/__https://journals.plos.org/digitalhealth/s/figures*loc-file-requirements__;Iw!!NnSTv5QBqPjS9UMk!LXzi5t0QgzWOMFCm-xWhA2cPfRcZFY-pyeVURkNHnxvvT07RjOxm-G4QSOG-MogUX4ks4KZy_u5h13ZVMuQ$)

We have generated separate figure files in .tif format.

4. Please insert an Ethics Statement at the beginning of your Methods section, under a subheading 'Ethics Statement'. It must include:

1) The name(s) of the Institutional Review Board(s) or Ethics Committee(s)
2) The approval number(s), or a statement that approval was granted by the named board(s)
3) (for human participants/donors) - A statement that formal consent was obtained (must state whether verbal/written) OR the reason consent was not obtained (e.g. anonymity). NOTE: If child participants, the statement must declare that formal consent was obtained from the parent/guardian.

We have now added the subheading ‘Ethics Statement’ (p. 6) at the beginning of the Methods section, stating that:

“*The study was approved by the University of Michigan Institutional Review Board (HUM00033029) and all subjects provided informed consent after receiving a complete description of the study.*”

5. We have noticed that you have uploaded Supporting Information files, but you have not included a list of legends. Please add a full list of legends for your Supporting Information files after the references list.

We have now included a list of legends for our Supporting Information file, with the section head “*Supporting Information Legends*”, after the References and Figures in the main text at p. 30.

6. If the reviewer comments include a recommendation to cite specific previously published works, please review and evaluate these publications to determine whether they are relevant and should be cited. There is no requirement to cite these works unless the editor has indicated otherwise.

Thank you for the reminder. There are no recommendations to cite any additional specific publications from the reviewers.

**Reviewers' Comments to the Author:**

Reviewer #1:

Dear Authors,

Thank you for your submission of the manuscript 'Patterns of smartphone typing performance by time awake: implications for unobtrusive ambulatory mental fatigue assessment.' The work is very interesting and depicts a novel form of assessing mental fatigue through use of smartphone keystrokes. The methods and results are very detailed.

Thank you.

The only minor revision is that there are some papers cited in the references which I cannot find cited in the text (27-30) which are relevant articles that I would have liked to see discussed either in the introduction's review of related works, or in the discussion.

Thanks for the thoughtful suggestion. We have incorporated reviews of these papers into the introduction and referenced them again in the discussion section. Please note that the reference numbers have changed following these revisions.

The related paragraph (p. 4, paragraph 2) in the Introduction now reads:

“*Recent research has investigated the connection between typing performance and mental fatigue, using both naturalistic and controlled environments to assess how cognitive functions influence typing speed and accuracy. Pimenta et al. proposed an unobtrusive method for measuring an individual’s level of fatigue through the interaction patterns of the individual with the mouse and keyboard of the computer.[5, 6] Al-libawy et al. proposed various methods to predict fatigue status using smartphone text entry performance metrics such as hold time and digraph time. [7. 8] de Jong et al. further reported that typing markers of computer keyboard could inform the mental fatigue status, by showing that decreased P3 brain potential amplitude (known to be related to mental fatigue [9-11] ) recorded by electroencephalography (EEG) is related to increase in typing time and typing errors.[12] Ross et al. discovered that typing speed on smartphones is positively associated with performances in the digital version of trail making test, a neuropsychological test that assesses visual attention, processing speed, and task switching.[13] Based on these validations of using typing performance as an indicator of mental fatigue, Jong et al. found that typing markers are susceptible to changes in behavior related to mental fatigue, with dynamic changes over different time scales such as time-on-task, time-of-day and day-of-week in an uncontrolled work environment.[14] A study of emergency medicine resident physicians demonstrated significant decrements in typing speed during night shift compared to day shift and evening shift.[15]These studies underscore the value of typing performance as a non-intrusive and reliable indicator for monitoring mental fatigue across various settings. However, these studies have been constrained by small sample sizes (all fewer than 100) and the need for a customized keyboard application to capture typing metrics, limiting the robustness of their conclusions and the utility and scalability of the tools utilized.*”

The related paragraph (p. 20, paragraph 1) in the Discussion reads:

“*This study has several strengths. First, the study benefits from high statistical power with longitudinal data collected over two months from more than 350 subjects, resulting in over 45,000 typing episodes, a much larger sample than previous studies on typing performance and mental fatigue. [5-8, 12-15]*”

Reviewer #2:

This paper benefits from several strengths, thereby making is complete and sound:
- The research question is very appropriate and interesting to address in a timely fashion
- The examined cohort is extensive and observed over a significant portion of time
- The statistical analysis is sound and particularly smart being based on a generalized estimating equation. This method is particularly appropriate for examining the relationship between typing and awake time since this equation typically estimates the parameters of a generalized linear model with a possible unmeasured correlation between observations from different time points, which is the case here
- The relationships between the independent and dependent variables are properly analyzed and discussed.

Thank you.

Tables 2 and 3 are challenging to browse in the submission as their indentation is fragmented. This should be improved in the final version with mid lines. When there is a significant p-value, maybe compute the effect size to determine the magnitude of the effect, which might be interesting since the sampling is extensive.

Thanks for pointing this out. We have now added mid-lines in **Tables 2 and 3**, and also used screenshots of the tables in the main text to ensure all the elements are on the same page, to improve the readability. The original tables were uploaded separately in excel files

Reviewer #3:

*Strengths*
This is an interesting and timely study that investigates the association between smartphone typing performance and time awake, with the broader goal of exploring whether typing behavior may serve as a passive indicator of mental fatigue. The scale of data collection is impressive—over 45,000 typing sessions from 366 medical interns—and demonstrates substantial effort and rigorous data handling. The statistical approach, including generalized estimating equations, GAM smoothing, and cosinor analysis, is methodologically sound and appropriate for modeling repeated measures in a real-world setting. The manuscript is clearly written, and the overall analytical framework is well-structured. This line of work has strong potential for digital-health applications, particularly in fatigue-sensitive occupations, though several aspects would benefit from clarification to ensure accurate interpretation of the findings.

Thank you.

*Weaknesses and suggestions*
1. While the study leverages “time awake” as an objective proxy for mental fatigue, it is important to note that time awake cannot fully replace or directly represent mental fatigue. Although the relationship between prolonged wakefulness and reduced alertness is well documented in prior literature, the study does not include any direct measurement of subjective or objective fatigue. Therefore, language implying fatigue detection, causal inference, or diagnostic capability should be avoided. The findings should be framed strictly as associations between typing performance and time awake.

We agree with the reviewer that time awake cannot fully represent mental fatigue. We completed a thorough check and confirmed that there’s no languages including ‘causal inference’, ‘diagnostic capability’ in the manuscript. All references to "association" in our findings strictly describe associations between typing performance and time awake.

There was one mention of ‘fatigue detection’, which we have now revised. The sentence (p. 4 paragraph 1) has been changed from:

“*Thus, current fatigue detection tools cannot be used for real-time, continuous detection of mental fatigue in the real world without interrupting regular activities.*”

to:

“*Thus, current tools cannot be used for real-time, continuous detection of mental fatigue signals in the real world without interrupting regular activities.*”

To ensure precision throughout the manuscript, we also edited several sentences which summarize our study, replacing ‘mental fatigue’ with ‘mental fatigue signals’:

p. 17, paragraph 2: “*In this study, we investigated the potential utility of real-time, unobtrusive and ambulatory smartphone keyboard typing performance metrics to continuously and passively measure mental fatigue signals.*”

p. 20, paragraph 1: “*First, the study benefits from high statistical power with longitudinal data collected over two months from more than 350 subjects, resulting in over 45,000 typing episodes, a much larger sample than previous studies on typing performance and mental fatigue signals.*”

2. Regarding typing behavior, several potentially important confounding factors were not included in the analysis. Although age, sex, device type, and time of day were appropriately adjusted for, medical interns’ typing performance is likely influenced by situational elements such as clinical workload, communication context, or multitasking demands. These real-world factors may partly explain why many prior studies were conducted in controlled laboratory environments, where such variability can be minimized. Although these contextual influences are difficult to capture in passive sensing data, they may meaningfully affect both typing speed and accuracy. A more explicit discussion of how unmeasured contextual factors could shape the observed associations would strengthen the interpretation of typing performance as a potential fatigue indicator.

In addition, presenting the frequency of low typing-performance z-score sessions occurring during periods when individuals typically perform well may help readers assess whether these sessions reflect meaningful variability or context-driven outliers.

Thanks for pointing this out. We now plot the proportion of typing sessions with typing speed z-score < -0.1 (corresponding to the red dashed line in Figure 3A) by hours awake (see below) and added it as Figure S8. The plot shows that: 1) the pattern matches the pattern of average typing speed presented in Figure 3A where the proportions of the low typing speed sessions were the lowest during 3 to 9 hours awake; 2) even at its minimum, the proportion remains around 40%, reflecting meaningful variability among the samples.

***Figure S8. Proportion of typing sessions with low speed by hours awake in the sample.*** *Low speed is defined as individual typing speed z-scores<0.1.*

We have also expanded the discussion of unmeasured contextual factors, to address this limitation more explicitly. In the discussion, we referred to the above Figure S8 to acknowledge the variations among the samples. Now it reads (p. 20 paragraph 2):

“*There are also some limitations to this study. First, there may be other confounding factors, such as caffeine and nicotine intake, medication, medical history, workload and demands, as well as motivation and context of typing, that could influence the associations between typing performance and time awake. For example, a higher workload may cause fatigue to accumulate more rapidly after shorter periods awake, while caffeine consumption may increase alertness even after extended wakefulness. Additionally, typing speed may differ between texting with friends and work-related emailing that requires greater cognitive effort. In fact, even during the periods associated with the highest average typing speed, there are still >40% typing sessions with individual typing speed z-scores below -0.1 (Figure S8), reflecting meaningful variability that may be driven by these contextual factors. However, these factors were not measured and thus not accounted for in our study. Future studies could benefit from systematically examining these contextual influences.*”

We also acknowledged the different individual patterns of the change of typing speed over the course of time awake, with two examples shown in Figure 4, as well as a paragraph discussing this matter in Discussion (p. 19, paragraph 2):

“*In addition to our findings related to the average trend among participants, we also found that there are individual differences in the relationship between typing speed and time awake. As illustrated in Figure 4, individuals differed in the timing and shape of their typing speed peaks and troughs with reference to time awake. Furthermore, meaningful departures from personal past typing speed trends can help to identify additional important driving factors. Specifically, continuous data collection enables establishment of personalized models that can be interrogated for associations with other individual differences (e.g., genotype, chronotype, type of work) and within-person predictors (e.g., exertion, breaks). Based on the results of the interaction tests, basic demographics like age and sex, as well as baseline and previous sleep duration, did not seem to be the factors moderating the relationship between time awake and typing speed. Future work is warranted in identifying between and within individual characteristics that may signal differences in mental fatigue development. From an applied perspective, tailored models could be developed to anticipate potential critical periods that maximize alertness and minimize fatigue for use in personalized feedback and intervention. Ongoing individualized monitoring of typing performance trends could also serve as a sentinel for unexpected critical drops in alertness.*”

3. The manuscript models both circadian timing and time awake, but partial collinearity between these variables may still concern readers—especially given that participants exhibit similar wake-up times (around 6 AM). The authors could strengthen the analysis by briefly reporting the correlation between time awake and time of day in this dataset, and by presenting a partial residual plot (from a GAM or GEE model) showing typing speed as a function of time awake after adjusting for time of day.

Thanks for the suggestion. We calculated the correlation between time awake and time of day (both sine and cosine form of its radian format), and added a sentence in Results (p. 12, paragraph 2) to briefly report their significant correlations:

“*Time awake was significantly correlated with both the sine (r = -0.46, p < 0.001) and cosine (r = 0.67, p < 0.001) transformations of time-of-day, i.e. the time awake tends to be longest near midnight (peak of cosine) and shortest in the morning (peak of sine).*”

The green line in Figures 3 shows the predicted typing speed as a function of time awake after adjusting for covariates including time of day, based on model (4) in the main text (p. 10, paragraph 3). Also, the figure legends included this sentence to clarify this:

“*The GAM-smoothed prediction lines from covariate-adjusted model (4) were plotted (green).*”

4. Finally, the interpretation of the deletion rate deserves clarification. Although statistically significant, the deletion rate shows minimal practical variation and appears much less sensitive to time awake than typing speed. Providing a concise explanation in the Results or Discussion—such as its low baseline variability, contextual dependence, or inherently smaller dynamic range—would make the rationale for focusing primarily on typing speed clearer.

Thanks for the suggestion. We have now expanded our discussion about the rate of deletion being relatively less sensitivity to time awake to include possible explanations (p. 19 paragraph 3), and now it reads:

“*In contrast to typing speed, typing accuracy, represented by the rate of deletion, remained relatively stable with time awake and time of day. This suggests that rate of deletion may not be as sensitive a measure of mental fatigue under the conditions studied. One possible explanation is that deletions during typing can reflect not just mistakes, but ongoing, deliberate editing, such as when composing charts or work emails, thus limiting its usefulness as a fatigue indicator. Additionally, autocorrection features on devices may further confound accuracy measures. Therefore, while typing accuracy seems less sensitive to time awake in this context, it warrants further exploration.*”

Reviewer #4:

Overall, the manuscript contributes valuable and novel insights with methodological rigor, but the authors should address and highlight data sharing limitations in accordance with PLOS policies. This will support the journal’s commitment to open science while respecting data privacy and third-party restrictions.

Thanks for the suggestion. Our data availability statement is:

“*SensorKit data cannot be shared publicly because of Apple's data sharing restrictions.*

*De-identified general study enrollment and survey data are available via the ICPSR repository (*[*https://www.openicpsr.org/openicpsr/project/129225/version/V1/view*](https://www.openicpsr.org/openicpsr/project/129225/version/V1/view)*).*

*De-identified wearable data is available through requests to the corresponding author. Reasonable requests will be fulfilled contingent on completion of a data use agreement with the University of Michigan.*”

We have now included this statement in the main text (p. 22), after Discussion and before the “Financial Disclosure Statement” section.

Reviewer #5:

The paper presents a study investigating the relationships between fatigue (measured as time from the last sleep episode) and speed and accuracy of typing on a smartphone in a large sample of MD interns, a population well-suited for a study of fatigue. The results show that typing speed and accuracy increase for the first 7 hours of waking time and decrease from hour 7 onwards, with speed of typing after 15 hours of waking time being more than 1 standard deviation lower than an individual average. The analysis of time of day and clarification of its additive effect to that of sleep duration is important for supporting the general conclusion and well-presented in Figure 5. This is a timely and insightful study and a well-written paper, I wholeheartedly support its publication as is.

Thank you.

Suggestions for improvement:
- Hours awake after about 18 yield a very low number of typing instances, and if all the instances are coming from the same few participants this could skew the results and the conclusions. An additional sensitivity analysis could limit the hours awake to 18 (inclusive). An additional plot could show the number of participants with at least one typing session for each hour (like Figure S2A, but with count of participants instead of events on the Y axis).

Thanks for the suggestions.

We conducted an additional sensitivity analysis restricting hours awake to a maximum of 18, which included 365 subjects and 43,413 typing sessions (compared to 366 subjects and 45,042 typing sessions in the full sample). The findings within the first 18 hours closely mirrored those observed in the full sample and are presented in Figure S4. We have also mentioned this additional analysis in the Methods and the Results.

***Figure S4. Average typing speed (A) and average rate of deletions (B) by number of hours awake, limited to a maximum of 18 hours awake.*** *Typing speed and rate of deletions were converted to individual z-scores. The error bars represent 95%CI. The GAM-smoothed prediction lines from model (4) were plotted (green). The Blue solid line represents the individual average (z-score = 0), while the red dashed line represents -0.1 standard deviation in (A) and +0.1 standard deviation in (B).*

Methods (p. 11 paragraph 2):

*First, to mitigate potential skewness arising from a small number of typing sessions occurring beyond 18 hours awake, we repeated the analysis by restricting to the typing sessions with a maximum of 18 hours awake.*

Results (p. 16 paragraph 2):

***Sensitivity Analyses: Restriction to a Maximum of 18 Hours Awake***
*There were 43,413 (96.4% of all 45,042) recorded typing sessions from 365 subjects (99.7% of all 366) that occurred within 18 hours awake. Analyses conducted on this subset yielded similar results (Figure S4).*

We also generated an additional plot showing the number of participants with at least one typing session for each hour awake, and added it to Figure S2. The figure now looks:

***Figure S2****. The numbers of: typing sessions by hours awake (A), subjects by hours awake (B), typing sessions by typing speed (C), and typing sessions by rate of deletion (D) in the sample.*

- In the formulae for computing Z-scores, I am not sure if the last subscript t (after the curly brackets) is needed, and if needed, shouldn’t it be t (not s) for the denominator in the deletion rate formula?

Thank you for catching this. The reviewer is correct that the *s* in the denominator in the deletion rate formula was a typo.

We used the annotation *t* to represent the date and time stamp rounded to the nearest half-hour, However, we appreciate that this annotation could benefit from clearer explanation. To address this, we have revised the Z-score equations for greater interpretability and added explanatory sentences to the text. The formulas section (p. 7 – p. 8), including both the Z-score equations and the preceding averaging equations, reads as follows:

“*The iPhone keyboard metrics from the first two months (July and August, 2023) of the intern year were included in the analysis. From the original measures of keyboard usage, we derived two metrics that reflect average typing performance for the multiple typing events (i) grouped by typing session at each day’s each half-hour grid point (t), where t represents the actual date and time stamp rounded to the nearest half-hour. All subjects share the same set of grid points; we calculate the typing metrics for subject s at t only if there is at least one associated typing session. First, the average typing speed for subject s at t was calculated by:*

<https://www.codecogs.com/eqnedit.php?latex=%5Ctext%7BTypingSpeed%7D_s(t)%20%3D%20%5Cfrac%7B%5Csum_i%20%5Cleft%5C%7B%5Ctext%7BtotalTypingDuration%7D_s(t%2Ci)%20%5Ctimes%20%5Ctext%7BtypingSpeed%7D_s(t%2Ci)%5Cright%5C%7D%7D%7B%5Csum_i%20%5Ctext%7BtotalTypingDuration%7D_s(t%2Ci)%7D#0>*.*

*Second, the average typing accuracy, represented by the rate of deletions, for subject s at t was calculated by:*

<https://www.codecogs.com/eqnedit.php?latex=%5Ctext%7BDeleteRate%7D_s(t)%20%3D%20%5Cfrac%7B%5Csum_i%20%5Ctext%7BtotalDeletes%7D_s(t%2Ci)%7D%7B%5Csum_i%20%5Ctext%7BtotalTaps%7D_s(t%2Ci)%7D#0>*.*

*Finally, because this study focuses on examining changes within individuals over the course of time since awake, typing speed and rate of deletions were converted to standardized z-scores for each subject s at t:*

<https://www.codecogs.com/eqnedit.php?latex=%5Ctext%7BTypingSpeed%20Zscore%7D_s(t)%20%3D%20%5Cfrac%7B%5Ctext%7BTypingSpeed%7D_s(t)%20-%20%5Cmu%5Cbig(%5Ctext%7BAllRecordedTypingSpeed%7D_s%5Cbig)%7D%7B%5Csigma%5Cbig(%5Ctext%7BAllRecordedTypingSpeed%7D_s%5Cbig)%7D%2C#0> *and*

<https://www.codecogs.com/eqnedit.php?latex=%5Ctext%7BDeleteRate%20Zscore%7D_s(t)%20%3D%20%5Cfrac%7B%5Ctext%7BDeleteRate%7D_s(t)%20-%20%5Cmu%5Cbig(%5Ctext%7BAllRecordedDeleteRate%7D_s%5Cbig)%7D%7B%5Csigma%5Cbig(%5Ctext%7BAllRecordedDeleteRate%7D_s%5Cbig)%7D#0>*,*

*where μ(.) and σ(.) represent the mean and the standard deviation of all records of typing speeds (*<https://www.codecogs.com/eqnedit.php?latex=%5Ctext%7BAllRecordedTypingSpeed%7D_s%20%3D%20%5Cleft%5C%7B%5Ctext%7BTypingSpeed%7D_s(t)%3A%20%5Ctextrm%7Bif%20subject%20s%20typed%20at%20t%7D%5Cright%5C%7D#0>*) or deletes rate (*<https://www.codecogs.com/eqnedit.php?latex=%5Ctext%7BAllRecordedDeleteRate%7D_s%20%3D%20%5Cleft%5C%7B%5Ctext%7BDeleteRate%7D_s(t)%3A%20%5Ctextrm%7Bif%20subject%20s%20typed%20at%20t%7D%5Cright%5C%7D#0>*) over all day all time points for subject s, respectively.*”

- For the typing statistics, was any typing included or only messaging and notes? E.g., typing in the browser, phone search, calendar, etc? This information could be included for clarity.

Thanks for giving us the opportunity to clarify. Any typing session was included for the typing statistics as there was no specific filtering for specific apps.

We have added this information in p. 5 paragraph 2 :

“*Specifically, the keyboard sensor captures the configuration of the phone’s keyboard and monitors its usage pattern, such as typing speed and accuracy, during the use of any App or other phone function, without recording the actual content typed or identifying the App or function.*”

- Inclusion of both sine and cosine for the time of day could be explained in a bit more detail. Why would both be included?

We include both sine and cosine terms because together they allow the model to capture any phase shift in a circular 24-hour rhythm. A single sine term assumes that the peak and trough occur at a fixed time of day, but in reality the daily pattern in typing performance may peak earlier or later. Including both sine and cosine terms lets the model estimate the amplitude (how big the daily ups and downs are) and the phase (the time of day the peak occurs) from the data itself. This is the standard approach in chronobiology and circular statistics because it gives the model the flexibility to match the real daily pattern instead of forcing a fixed shape.

To make it clearer, we edited the related sentences in the Methods (*p. 10, paragraph 2*):

“*The second model examined this relationship after adjusting for covariates, including age, biological sex, time of day (modeled using the sine and cosine of its radian format to capture the circular nature of time and flexibly represent rhythms of any phase and amplitude),...* ”
